# Weight Development in Children After Gastric Bypass Surgery 

**Published:** 2019-12

**Authors:** Linda Larsson, Mona Landin-Olsson, Charlotta Nilsson

**Affiliations:** 1 Department of Paediatrics, Institution of Clinical Sciences, Helsingborg Hospital, Lund University, Sweden; 2 Department of Endocrinology, Institution of Clinical Sciences, Skåne University Hospital, Lund University, Sweden

**Keywords:** Child, Weight Development, Gastric Bypass

## Abstract

**Objective:** More and more young obese women get pregnant after undergoing gastric bypass surgery (GBP) but little is known about weight development in their offspring. The first aim of this study was to investigate weight development of children whose mothers have undergone GBP before pregnancy and compare them to age specific reference values in Sweden. Second aim was to study the frequency of small for gestational age (SGA) in this population.

**Materials and methods:** Weight of offspring (38 male and 28 female) where the mother had undergone GBP before pregnancy were studied from birth up to 18 months of age and compared to age-specific reference values in Sweden.

**Results:** The boys to mothers who had undergone GBP before pregnancy weighed more than Swedish reference values at 6 months, 8.44 ± 1.18 kilogram (kg) (n = 35) vs. 7.98 ± 0.81 kg (n = 1388; p = 0.001), and less at 18 months, 11.54 ± 0.93 kg (n = 19) vs. 12.27 ± 1.19 kg (n = 862; p < 0.001). The girls to mothers who had undergone GBP before pregnancy weighed more than Swedish reference values at 6 months, 7.84 ± 1.00 kg (n = 28) vs. 7.50 ± 0.77 kg (n = 1375; p = 0.020). Frequency of SGA was 3.0%.

**Conclusion:** No clear pattern was found concerning the short-term weight development of the children. However, studies with larger material and more follow up time must be performed.

## Introduction

Obesity has become an epidemic around the world and WHO estimated that in 2014 over 13% of all adults in the world were obese (about 600 million adults). The prevalence of obesity have more than doubled since 1980. For children the prevalence of overweight/obesity under the age of five was 6.3 % in 2013 (1). In Sweden around 10-12% of the population over 16 years of age and about 2% of children were obese in 2012 (2). Besides negative health impact from obesity, for women there is an increased risk for pregnancy complications and infertility (3). When attempts to a healthier lifestyle with calorie restricted food intake and exercise is not enough surgery such as laparoscopic Roux-en-Y gastric bypass (GBP) can be considered (4). The procedure has been shown to result in good and maintained weight loss for a majority of patients with morbid obesity (5). Weight loss for women also improves fertility and decreases the risk for diabetes, hypertension and preeclampsia during pregnancy (6). 

Offspring of women who have undergone GBP before pregnancy have an increased incidence of being small for gestational age (SGA) and of intrauterine growth restriction (IUGR) (7, 8). The long-term effects on these children however have been poorly studied. One study where prevalence of obesity in children aged 2-18 years born to women before or after they underwent their GBP surgery showed 52% lower obesity rate in the children born after GBP surgery compared to their sibling born before GBP surgery. No difference compared with general population was found (9). However, more studies are needed to examine possible long-term effects in the offspring of women who have undergone GBP. 

First aim of this study was to investigate weight development of children whose mothers have undergone GBP before pregnancy and compare them to age specific reference values in Sweden. Second aim was to study the frequency of SGA in this population.

## Materials and methods

In the district of Lund, Sweden, there were 206 pregnancies during 2012-2015 where the women previously had undergone GBP. Before 2012 very few women in our region underwent GBP and therefore this year was chosen as first year of the study. The study was approved by the Ethical Board at Lund University, Sweden (746/2014, 23/2016) and in accordance with the Helsinki declaration.

All women were contacted by letter asking for permission from both parents to collect and study weight- and height-charts of their children from their Health Care Centre. 

In total, 55 parents gave their written consent, yielding a total of 66 children participating in the study.

Data from weight- and height-charts from all children participating in the study were collected for weight at birth, at 3, 6, 12 and 18 months, all per the routine measurements in Sweden. Birth weight was measured by midwifes at delivery and the other weights at their Health Care Centres. Frequency of SGA (birth weight < 2SD) was noted at birth (10). Very few heights were available and therefore weights were used in this study for comparisons. Weights of the children, male (n = 38) and female (n = 28) separately, at birth, 3, 6, 12 and 18 months of age were compared to the population based age specific reference values in Sweden consisting of 3650 full-term healthy Swedish children born between 1973 and 1975 (11). There were 13 premature children born < 37 week and for these children age-transformed premature curves were used (11, 12). Because not all children had reached the age of two when the data was collected and some data were missing from the records there are some missing values in the results. 

***Statistical analysis*** ***:*** The D’Agostino-Pearson test was used to examine whether the data were normally distributed. Results are presented as mean ± standard deviation (SD) and T-test was used for comparison between groups. Frequencies are presented as number and percent (%). The program MedCalc® for Windows (version 13.1.2.0) was used for the statistics. The program GraphPad Prism was used to make graphs. A p-value of < 0.05 was considered significant.

## Results

**Figure 1:**
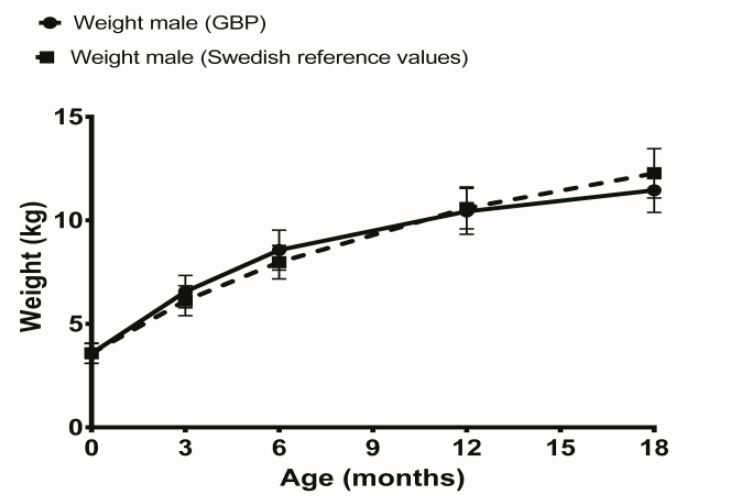
Weight development in male offspring’s to women with previous gastric bypass (GBP) compared to Swedish reference values.

**Figure 2:**
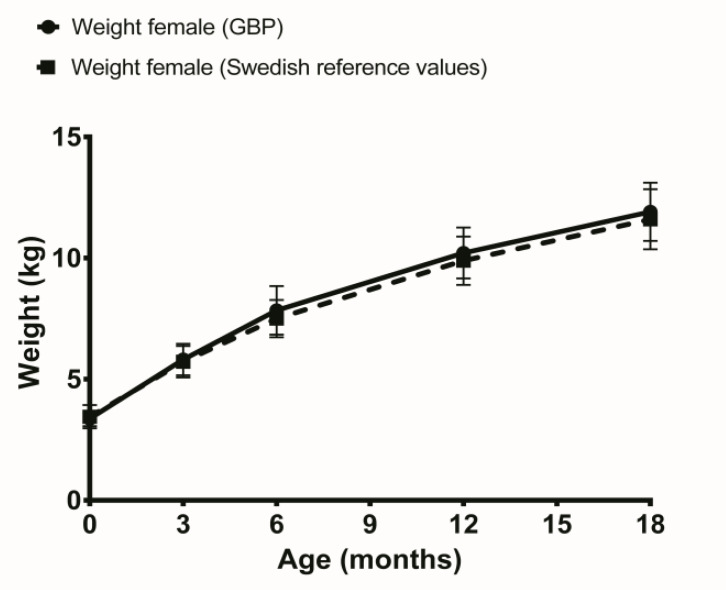
Weight development in female offspring’s to women with previous gastric bypass (GBP) compared to Swedish reference values.

Comparisons between weights of boys after GBP pregnancy and Swedish reference values showed significantly higher weight at 6 months of age in the GBP pregnancy group but significantly less weight at 18 months, shown in table 1 and illustrated in figure 1. The same comparisons for girls showed significantly higher weight at 6 months of age in the GBP pregnancy group compared to Swedish reference values, shown in table 1 and illustrated in figure 2.

Among the 66 children born after GBP surgery two children (3.0%) were SGA.

## Discussion

In this study we wanted to study weight development of offspring to women who had undergone GBP surgery before pregnancy as well as frequency of SGA in order to examine the impact of GBP.

**Table 1:** Comparison between weights of children (kg) where the mother had undergone GBP and Swedish reference values

**Gender**	**Age (months)**	**Weight of all children after ** **GBP pregnancy (kilogram)**	**Weight of Swedish reference ** **values (kilogram)**	**P-value**
Male	0	3.50 ± 0.49 n = 37	3.58 ± 0.49 n = 1760	0.314
Male	3	6.36 ± 0.96 n = 37	6.12 ± 0.72 n = 1471	0.051
Male	6	8.44 ± 1.18 n = 35	7.98 ± 0.81 n = 1388	0.001
Male	12	10.21 ± 0.91 n = 25	10.60 ± 1.01 n = 787	0.055
Male	18	11.54 ± 0.93 n = 19	12.27 ± 1.19 n = 862	0.008
Female	0	3.37 ± 0.31 n = 28	3.45 ± 0.48 n = 1724	0.404
Female	3	5.81 ± 0.65 n = 28	5.72 ± 0.65 n = 1449	0.454
Female	6	7.84 ± 1.00 n = 28	7.50 ± 0.77 n = 1375	0.020
Female	12	10.21 ± 1.05 n = 24	9.89 ± 1.00 n = 821	0.119
Female	18	11.91 ± 1.20 n = 16	11.60 ± 1.24 n = 846	0.323

The data is presented as mean ±SD.

All women in the study came from the same area in Sweden and had undergone the same GBP surgery and therefore this material is a representative sample of this group of women and children. Weight of the children was collected from Health Care Centres where it was correctly measured which ensures validity of the material. 

The boys weighed more than the Swedish reference values at 6 months of age and weighed less than reference values at 18 months of age. For girls born after GBP surgery, the same pattern was shown at 6 months of age. Previous study from Australian pregnant women who had previous undergone GBP did not find any differences in birth weight compared to normal community birth weights (13). 

One limitation of this study was that we only investigated the offspring’s weight up to 18 months of age. It is possible that the weight development would difference more from the Swedish reference values when studied over a longer time period. Another limitation is that the Swedish reference values are based on an older material with children born 1973 to 1975 (11) whereas the children in our study were born from a different time period, 2012-2015. However this would probably give a bias towards greater difference since children weigh more nowadays (1). Even though the environment play a role in the utero, effect with higher risk for IUGR because of the GBP surgery is as least as important (8), but unfortunately we do not have that information in this study. 

In the future we would like to follow the offspring’s weight and height development over a longer time period since we only have data since 2012. Then more of the children would be over two years of age and we would also be able to use BMI instead and calculate frequency of overweight and obesity (14). We would also like to compare children born to the same women before GBP pregnancy. Previous studies regarding this are inconclusive. Some studies have shown significantly lower prevalence of obesity among children born after their mother had undergone GBP than among their siblings born before (15) whereas other have shown decrease in overweight but not obesity (16) or no significant changes in BMI (17). 

The prevalence of SGA among newborns in this study was 3.0%. According to the National Board of Health and Welfare the prevalence of SGA among newborns in Sweden was 2.4% during 2012-2014 (18). Our study is small but still differs from the common understanding that previous GBP is associated with higher risk of SGA. A Swedish study published in 2013 showed an increased risk of SGA at 5.2% in women with previous GBP (19). A Danish study from the same year showed a 7.1% prevalence of SGA (20). The low percentage of SGA in this study can be a result of the women being sufficiently substituted with essential nutrients after the GBP.

## Conclusion

In this small material no clear pattern was found concerning short-term weight development of the children. More studies with larger material and more follow up time must be performed.
